# Flavor compounds regulation in rice-flavor Baijiu by *Bacillus velezensis*: a genetic and functional analysis

**DOI:** 10.3389/fmicb.2026.1891609

**Published:** 2026-07-24

**Authors:** Hong Ren, Ren Huang, Yingying Xie, Qinghua Chen, Feng Chen, Runhua Huang, Yushi Liang, Haiyan Lu, Jianwei Zheng, Guidong Huang, Xianfeng Zhong

**Affiliations:** 1 College of Food Science and Engineering, Foshan University, Foshan, Guangdong, China; 2 Guangdong Traditional Fermented Food Engineering Technology Research Center, Foshan, China; 3 Guangdong Province Food Circulation Safety Control Engineering Technology Research Center, Foshan, China; 4 Foshan Brewing Engineering Technology Research Center, Foshan, China; 5 Foshan Agricultural Biological Manufacturing Engineering Technology Research Center, Foshan, China

**Keywords:** *Bacillus velezensis*, genome analysis, rice-flavor Baijiu, strain specificity, volatile flavor compounds

## Abstract

To achieve targeted enhancement of the flavor complexity of rice-flavor Baijiu, this study evaluated the impact of fortified fermentation with four saccharification-promoting *Bacillus velezensis* strains on the volatile profile of fermented grains (Jiupei), and further dissected the genetic basis of the key strains. The volatile composition of Jiupei was analyzed using headspace solid-phase microextraction gas chromatography–mass spectrometry (HS-SPME-GC–MS) coupled with multivariate statistical analysis to assess the effect of *B. velezensis* bioaugmentation on flavor. All four fortified fermentation groups upregulated the contents of 304 volatile compounds, including 78 terpenoids and 62 esters. Using |lg FC| ≥ 1 and *p* < 0.05 as screening criteria, 68 significantly differential metabolites were identified, such as *β*-cubebene, ethyl 2-methyl-2-butenoate, and benzyl benzoate. Among the compounds with relative odor activity values (rOAV) ≥ 1, strains FUBL1 and FUBL3 significantly increased the level of 4-vinylguaiacol, and FUBL3 specifically elevated the content of farnesylacetaldehyde; together, these flavor substances constructed a rich Baijiu flavor profile. Comparative whole-genome analysis revealed that FUBL1 and FUBL3 harbor a complete methylerythritol phosphate (MEP) pathway for terpenoid biosynthesis as well as key genes for ester precursor synthesis. Notably, FUBL3 uniquely possesses a citronellol/citronellal dehydrogenase gene (*atuB*) and a flavin prenyltransferase gene (*ubiX*), which likely correlates strongly with its greater terpenoid diversity. In summary, this study elucidates the mechanism by which *B. velezensis* regulates the formation of the characteristic flavor of rice-flavor Baijiu from the perspectives of flavor component profiling and genomics, providing a theoretical basis for the mining of functional microbial resources and their precise manipulation in Baijiu brewing.

## 1 Introduction

Rice-flavor Baijiu produced in the Lingnan region of China is made from rice as the raw material, with Xiaoqu (a traditional starter) serving as the saccharifying and fermenting agent. The brewing process employs an initial solid-state cultivation and saccharification stage, followed by a semi-solid-state alcoholic fermentation, and is then completed through distillation, aging, and blending (Yu et al., 2021). Owing to the single raw material and short fermentation period, the types and quantities of flavor compounds generated are relatively limited, leading to low flavor complexity (Qu et al., 2023; Liang et al., 2025; Jin et al., 2017; Yin et al., 2020). This results in a significant difference from other Baijiu aroma types, but also leads to serious product homogeneity and fierce market competition. With the upgrading of consumption and the transformation of product structures, alcoholic beverage consumption has increasingly exhibited trends toward lower alcohol content, health-consciousness, and personalization (Li et al., 2024). However, when the alcohol content of rice-flavor Baijiu is reduced, its flavor quality tends to decline accordingly (Gu et al., 2024). At present, the overall content of flavor compounds in rice-flavor Baijiu is generally low, resulting in a relatively thin flavor profile, as well as frequent bitter aftertastes and post-drinking headaches (Qu et al., 2023). This problem is closely associated with the microbial community composition and diversity during the brewing process (Hou et al., 2025; Zou et al., 2023). Therefore, it is of great significance to elucidate the functional mechanisms of key microorganisms in flavor formation and to promote the transition of the brewing system from reliance on unstable natural microbial communities to targeted regulation based on well-characterized functional microorganisms, thereby realizing the stable production of high-quality rice-flavor Baijiu.

Studies have demonstrated that fortified fermentation with functional microorganisms is an effective strategy for enriching volatile flavor metabolites in Baijiu and improving its quality (Wang et al., 2017; Udomsil et al., 2017). Bacteria represent one of the core microbial groups in alcoholic beverage fermentation. Through diverse metabolic pathways, they produce flavor compounds such as organic acids and esters, thereby exerting a profound influence on the overall flavor profile of the liquor (Liu and Sun, 2018). The genus *Bacillus* constitutes a key functional microbial group in fermented grains. *Bacillus* species secrete a variety of hydrolytic enzymes that degrade starch, proteins, and other macromolecules, thus playing a vital role in Baijiu fermentation (Li et al., 2019). In particular, *Bacillus velezensis* is widely distributed in traditional fermentation systems and has been applied to fortify Baijiu fermentation, enhancing the production of flavor compounds and the overall quality (Tong et al., 2024; Mi et al., 2025). Previous studies have demonstrated that Cheng and Jiang et al. used a high tetramethylpyrazine (TTMP)-producing *B. velezensis* strain in intensified fermentation, significantly increasing the contents of total esters and TTMP in Baijiu and improving its flavor (Cheng et al., 2026; Jiang et al., 2025). Through multi-omics approaches, Wang dissected how microbial communities shape flavor and found that co-cultivation of *B. velezensis* with *Saccharomyces cerevisiae* markedly elevated the levels of long-chain fatty acid esters, such as ethyl decanoate and ethyl laurate (Wang et al., 2026). Beyond modulating the abundance of the microbial community, *B. velezensis* also enhances the saccharification and liquefaction capacities of Daqu and increases the contents of both volatile and non-volatile metabolites (Zhang et al., 2024). Therefore, elucidating the functional mechanisms by which key microorganisms contribute to flavor formation, and promoting the transition of brewing systems from dependence on unstable natural microbial consortia toward targeted regulation based on well-defined functional microorganisms, are of great significance for achieving the consistent production of high-quality rice-flavor Baijiu.

Currently, there are few reports on *Bacillus velezensis* in rice-flavor Baijiu. To address this gap, we used *Bacillus velezensis* strains with strong degradation activity, previously screened in our laboratory, to conduct simulated fermentation experiments. The volatile components of the fermented grains were subjected to metabolomics analysis using headspace solid-phase microextraction coupled with gas chromatography–mass spectrometry (HS-SPME-GC–MS). By integrating the relevant metabolic genes of these strains, we elucidated the key pathways for the synthesis of flavor metabolites, aiming to provide a theoretical basis for the optimization of the flavor quality of rice-flavor Baijiu and the development and application of functional strains.

## 2 Materials and methods

### 2.1 Strains and materials

*Bacillus velezensis* strains FUBL1, FUBL2, FUBL3, and FUBL10 were independently isolated by our research group from glutinous rice wine mash collected from a distillery in Guangdong, China. Experimental validation demonstrated that FUBL1 and FUBL3 promote saccharification and fermentation during the production of rice-flavor Baijiu. *Bacillus velezensis* FUBL3 has been granted a Chinese national invention patent (Patent No. ZL202511285101.2) and has been deposited at the China General Microbiological Culture Collection Center (CGMCC) under accession number CGMCC No. 64133.

The Jiuqu (traditional fermentation starter) used in this study was provided by a distillery in Foshan City, Guangdong Province, China. Ordinary rice was used as the raw material for fermentation.

### 2.2 Instruments and equipment

Agilent 8,890-7000D gas chromatograph-mass spectrometer; Retsch MM400 mixer mill; CTC Analytics AG SPME Arrow solid-phase microextraction device; Agilent 120 μm DVB/CWR/PDMS extraction fiber; Agilent DB-5MS (30 m × 0.25 mm × 0.25 μm) column; CTC Analytics AG Agitator sample heating chamber; GR60DA fully automatic autoclave; LRH-150 biochemical incubator; SW-CJ-1FD clean bench; ALLEGRA-64R high-speed refrigerated centrifuge.

### 2.3 Fermentation experiment

Bacterial culture preparation: A glycerol stock of *Bacillus velezensis* was inoculated at 1% (v/v) into LB liquid medium and incubated at 37 °C with shaking at 160 rpm for 12 h. This seed culture was then transferred at 2% (v/v) into fresh LB liquid medium and incubated under the same conditions (37 °C, 160 rpm) for 4–5 h. The resulting bacterial suspension, with a cell density of 10^6^–10^7^ CFU/mL, was used as the inoculum for fermentation.

Rice (4 kg) was precisely weighed, mixed with 3.6 kg of water, and steamed until fully cooked. After cooling to approximately 35 °C, Jiuqu (0.8% of the rice weight) and bacterial liquid (15% of the rice weight) were added and mixed thoroughly. The blank control group received the same amount of Jiuqu and an equal volume of ddH_2_O. The mixture was then transferred into a fermentation tank for fermentation. Following a 24 h saccharification period, water equal to the weight of the original rice was added, and fermentation was continued for 21 days. An appropriate aliquot of fermentation broth was centrifuged at 8 °C and 5,000 rpm for 10 min. The supernatant was stored at −20 °C (freezer) until flavor compound analysis (Tang et al., 2022; Zheng et al., 2016).

### 2.4 Conditions for headspace solid-phase microextraction

The fermented grains samples were retrieved from the −80 °C freezer and ground in liquid nitrogen, followed by thorough vortex mixing. For solid samples, 500 mg was weighed into a headspace vial; for liquid samples, 1 mL was transferred into the vial. Subsequently, saturated NaCl solution and 20 μL of an internal standard solution (10 μg/mL) were added. Extraction was performed using fully automated headspace solid-phase microextraction.

The sample was maintained at a constant temperature of 60 °C and agitated for 5 min. A 120 μm DVB/CWR/PDMS solid-phase microextraction (SPME) fiber was then inserted into the headspace vial for extraction over a period of 15 min. Following extraction, the fiber was desorbed at 250 °C for 5 min, after which the analytes were separated and identified by gas chromatography–mass spectrometry (GC–MS). Prior to extraction, the SPME fiber had been conditioned at 250 °C for 5 min in a fiber conditioning station.

### 2.5 Conditions for gas chromatography–mass spectrometry

ADB-5MS capillary column (30 m × 0.25 mm × 0.25 μm; Agilent J&W Scientific, Folsom, CA, USA) was used with high-purity helium (≥ 99.999%) as the carrier gas at a constant flow rate of 1.2 mL/min. The injector temperature was set at 250 °C, and samples were introduced in splitless mode with a solvent delay of 3.5 min. The temperature program was as follows: hold at 40 °C for 3.5 min; increase to 100 °C at 10 °C/min; then raise to 180 °C at 7 °C/min; finally ramp to 280 °C at 25 °C/min and maintain for 5 min.

Electron ionization (EI) was employed with the ion source temperature maintained at 230 °C, the quadrupole temperature at 150 °C, and the MS interface temperature at 280 °C. The electron energy was set to 70 eV. Detection was performed in Selected Ion Monitoring (SIM) mode, allowing for targeted analysis of qualitative and quantitative ions with precise scanning.

### 2.6 Whole-genome sequencing and functional annotation of strains

Cells of *Bacillus velezensis* strains FUBL1 and FUBL3 at the logarithmic growth phase were harvested, flash-frozen in liquid nitrogen, placed in an insulated container with dry ice, and shipped to MAGIGENE Company (Guangzhou, Guangdong, China) for whole-genome sequencing. Complete genome sequences were assembled *de novo* from the third-generation sequencing reads using Unicycler v0.4.8. Coding sequences (CDSs) in the genomes were predicted and analyzed using Prokka and other bioinformatics tools. The predicted gene sequences were subsequently searched against the KEGG database^1^ and other databases with an E-value threshold of ≤ 1 × 10^−5^ for gene annotation. The whole-genome sequences of FUBL1 and FUBL3 have been deposited in the National Microbiology Data Center (NMDC).^2^

### 2.7 Data processing

Raw mass spectrometry data were processed using MassHunter software for qualitative and quantitative analyses. Metabolomics data were analyzed with Progenesis QI v3.0 (Waters Corporation, Milford, USA) for metabolite identification. Principal Component Analysis (PCA), Orthogonal Partial Least Squares-Discriminant Analysis (OPLS-DA), and cluster analysis were performed using R software.^3^ Bar charts were generated with OriginPro 2025.

## 3 Results and analysis

### 3.1 Classification of volatile metabolites in Jiupei (fermented grains)

**Figure 1:**
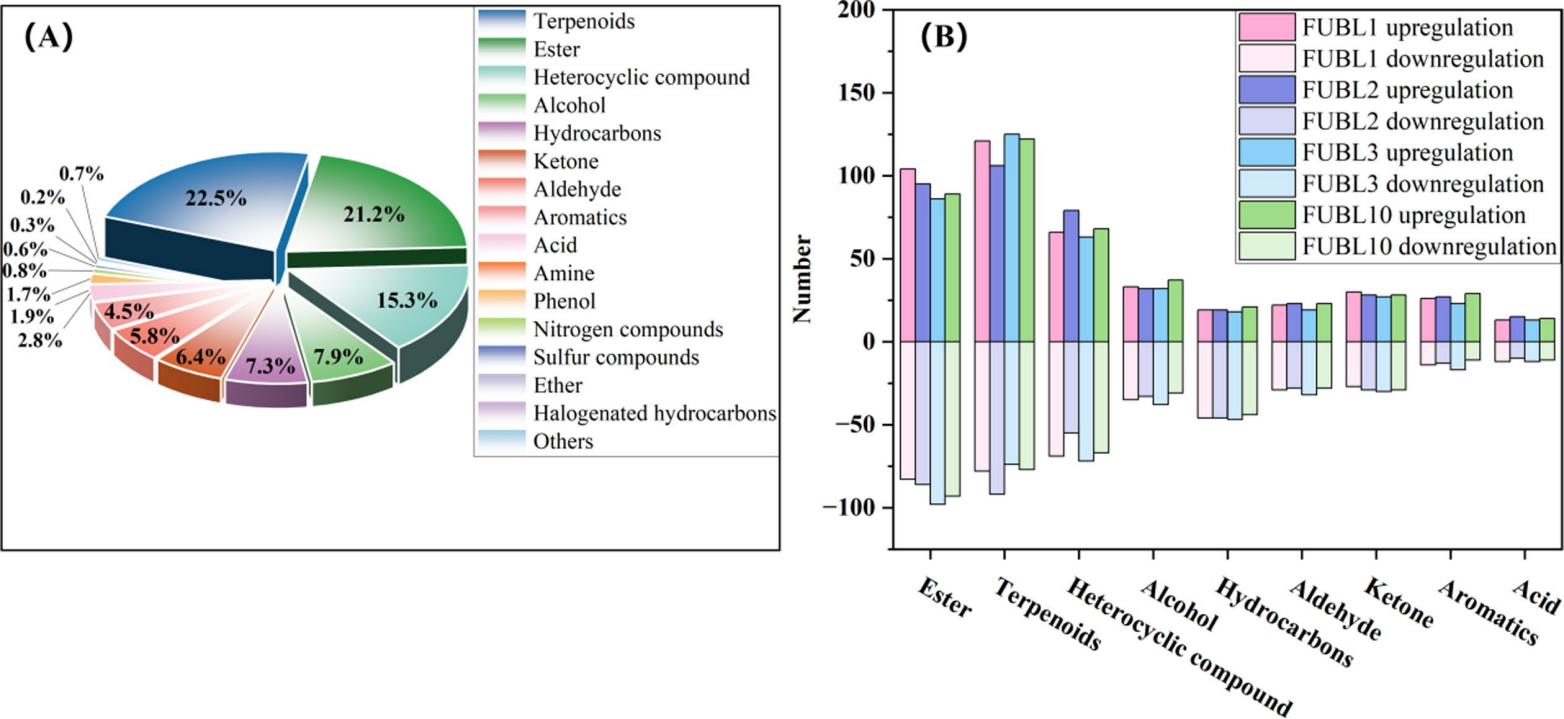
Classification of volatile metabolites in fermented grains (Jiupei). **(A)** Composition of flavor metabolite categories. **(B)** Number of differentially accumulated metabolites between groups.

The volatile metabolites in the fermented grain samples were analyzed by HS-SPME-GC–MS, and the results are presented in Figure 1 (see Supplementary Figure 1). A total of 885 volatile metabolites were detected across all fermented grain samples, including 198 terpenoids, 188 esters, 135 heterocyclic compounds, 70 alcohols, 65 hydrocarbons, 57 ketones, 51 aldehydes, 40 aromatics, 25 acids, 17 amines, 16 phenols, 7 nitrogen compounds, 5 sulfur compounds, 3 ethers, 2 halogenated hydrocarbons, and 6 others. In the fortified fermentation groups, terpenoids, esters, and heterocyclic compounds were the most abundant classes in terms of the number of species. Compared with the blank control group, the FUBL1 and FUBL3 groups exerted the most significant effects on the number of ester species, while the FUBL3 and FUBL2 groups exhibited the most pronounced influences on the numbers of terpenoid and heterocyclic compound species. Taken together, among the four fortified fermentation groups, the FUBL3 group showed the most significant impact on the number of key flavor compounds, particularly esters and terpenoids.

Terpenoids and esters are the predominant metabolites in the fermented grains of rice-flavor Baijiu, followed by heterocyclic compounds, alcohols, ketones, and aromatics, all of which play an important role in shaping the taste, aroma, and flavor of the liquor. The abundant terpenoids in Baijiu generally originate from two major sources (Yang et al., 2025): first, terpenoids inherently present in the raw brewing materials are carried over into the base liquor during fermentation and distillation. Second, they are produced through microbial metabolism, involving microorganisms such as actinomycetes and *Saccharomyces cerevisiae*. Most terpenoids produce strong and pleasant olfactory impressions, exhibiting floral, woody, and fruity aroma characteristics; for instance, limonene possesses a typical citrus and lemon-like aroma (Wu et al., 2015). Although terpenoids occur only in minor quantities in alcoholic beverages, they are nonetheless critically important for flavor development. In wine, for example, linalool, geraniol, cis-geraniol, and citronellol are the principal terpenoids responsible for flavor contribution (Wang Q. et al., 2025). Moreover, terpenoids also exhibit a variety of biological activities, including antibacterial, antiviral, antioxidant, and pro-digestive effects (Li et al., 2025; Zhang et al., 2021). Furthermore, esters, aromatic hydrocarbons, and other compounds in the fermented grains (Jiupei) are key contributors to the flavor profile of Baijiu, originating primarily from microbial fermentation and metabolism, the Maillard reaction, and enzymatic reactions (Xu et al., 2022). Esters mainly impart fruity, floral, sweet, creamy, and other flavor characteristics to Baijiu. Because most esters possess low odor thresholds, they play a crucial role in the formation of rice-flavor Baijiu flavor. Differences in the types and concentrations of esters among different Baijiu products endow them with unique flavor characteristics (Liang et al., 2024).

### 3.2 Flavor metabolite grouping analysis

Principal component analysis (PCA) and orthogonal partial least squares discriminant analysis (OPLS-DA) were applied to the volatile flavor compounds detected in the Jiupei (fermented grain) samples (The results are presented in Supplementary material 1). The PCA results revealed that PC1 and PC2 explained 40.37 and 24.68% of the total variance, respectively, and the four groups bioaugmented with different *Bacillus velezensis* strains were clearly separated from the blank control group in the score plot. This indicates that different *B. velezensis* strains exerted profoundly different effects on the formation of flavor compounds in the fermented grains, with significant strain-specific differences. For the OPLS-DA model (Yun et al., 2021), R^2^ = 0.991 and Q^2^ = 0.652, both exceeding 0.5, demonstrating a reliable model fit and good predictive capability for exploring the influence of *B. velezensis* enhanced fermentation on the flavor compounds in rice-flavor Baijiu.

Based on the OPLS-DA model, the influence of four *Bacillus velezensis* strains on the composition of flavor metabolites during rice-flavor Baijiu fermentation was systematically analyzed. The results showed that the four strains jointly increased the contents of 304 flavor metabolites, including 78 terpenoids such as trans-geranylgeraniol, (−)-*γ*-elemene, and *α*-pinene; 62 esters such as hexanedioic acid dimethyl ester, octanoic acid 2-furanylmethyl ester, and hexanoic acid butyl ester; 49 heterocyclic compounds such as indole and theophylline; and 11 phenolic compounds such as guaiazulene. For example, octanoic acid 2-furanylmethyl ester imparts fruity, roasted, and sweet notes to the liquor body (Wang R. et al., 2025), while hexanoic acid butyl ester contributes not only fruity and sweet notes but also pleasant aromas such as floral and creamy scents (Wang Y. et al., 2024). These compounds constitute the common flavor basis of the fortified fermentation groups. Further analysis revealed that different strains exerted strain-specific effects on flavor metabolites.

Compared with the blank control group, in the FUBL1 group, 121 terpenoids, 105 esters, and 66 heterocyclic compounds were upregulated; among them, 11 flavor metabolites, including butanoic acid 2-methyl-2-methylbutyl ester, hydrocoumarin, and *β*-eudesmol, were unique to this group. In the FUBL2 group, 106 terpenoids, 96 esters, and 79 heterocyclic compounds were upregulated, and 51 compounds, such as citronellyl isobutyrate, aromandendrene, and 1,3-dihydro-2H-indol-2-one, were specific to this group. In the FUBL3 group, 125 terpenoids, 87 esters, and 63 heterocyclic compounds were upregulated, with 6 metabolites, including alloaromadendrene and *α*-terpinyl acetate, being uniquely present. In the FUBL10 group, 122 terpenoids, 90 esters, and 68 heterocyclic compounds exhibited elevated abundance, and 17 flavor substances, such as butanoic acid 2-pentenyl ester and caryophyllenyl alcohol, were specific to this group. In addition, certain flavor metabolites displayed synergistic expression patterns across groups: for example, trans-trans-farnesal, 3-phenyl-2-propen-1-ol, and butanoic acid 2-methyl-ethyl ester were upregulated only in the FUBL1 and FUBL3 groups, whereas benzeneacetic acid ethyl ester and isopinocarveol were elevated only in the FUBL2 and FUBL10 groups.

**Figure 2:**
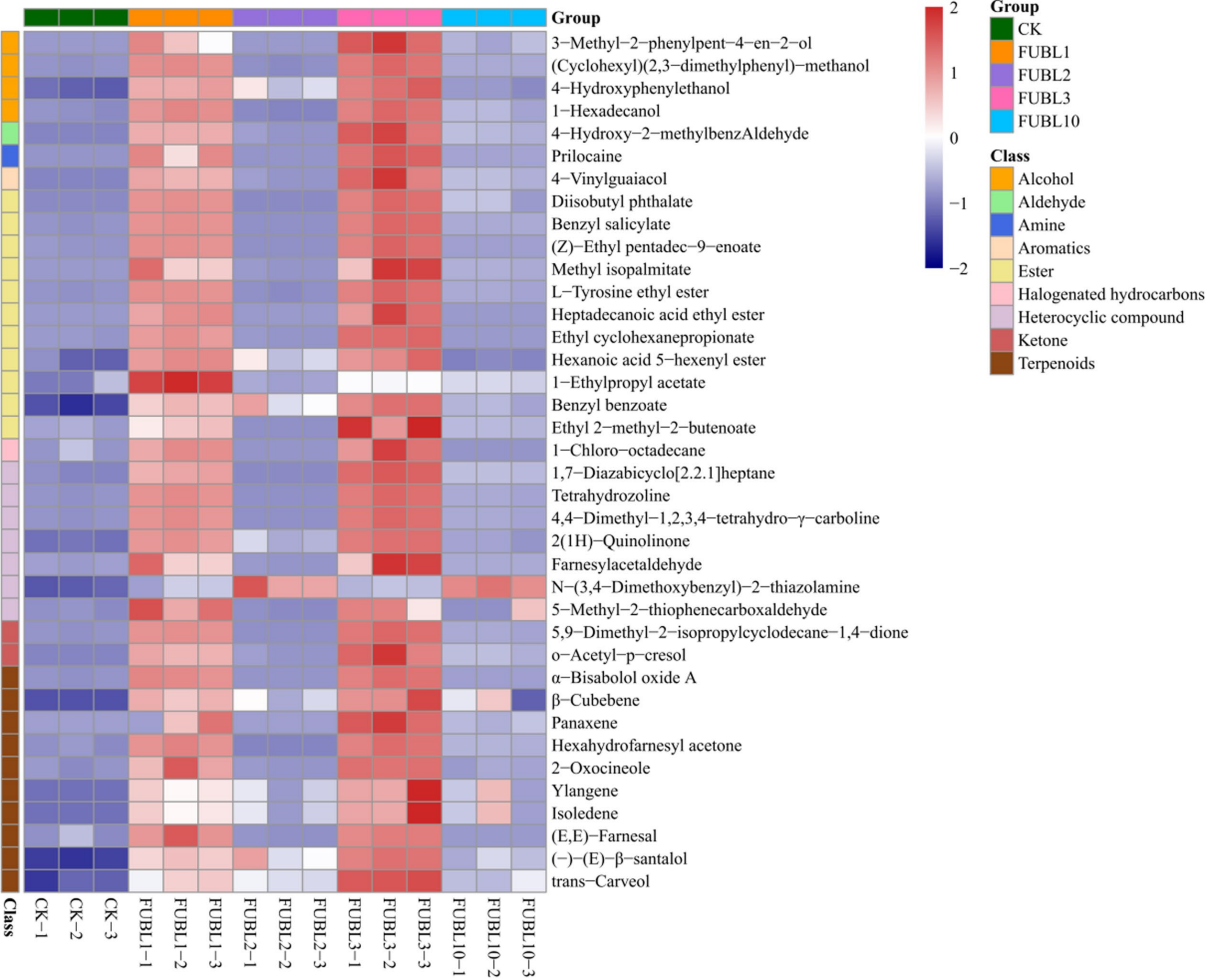
Heatmap of differential metabolites among groups.

Based on the screening criteria of |lg FC| ≥ 1 and *p* < 0.05, a total of 68 significantly differential metabolites were identified, as shown in Figure 2 (Substances with elevated levels are indicated in Supplementary Figure 1, whereas those with reduced levels are presented in the Supplementary material.). Among the four *Bacillus velezensis* strains used for fortified fermentation, the only differentially abundant flavor metabolite jointly promoted by four strains was *β*-cubebene, which contributes woody, fruity, and citrusy notes to the liquor. In the FUBL1 group, 33 flavor metabolites including benzyl salicylate, 4-vinylguaiacol, and β-cubebene were significantly upregulated, whereas 13 metabolites such as 1-butanol-2-methyl-acetate were downregulated; compared with the blank control group, the content of β-cubebene increased by 63 fold and that of benzyl salicylate increased by 56 fold. In the FUBL2 group, 3 flavor metabolites including β-cubebene were significantly upregulated, and 12 metabolites such as benzeneacetic acid were downregulated; compared with the blank control group, the β-cubebene content increased by 33 fold and the p-hydroxyphenyl methyl carbinol content increased by 14 fold. In the FUBL3 group, 36 flavor metabolites including ethyl 2-methyl-2-butenoate, benzyl benzoate, and (−)-(E)-β-santalol were significantly upregulated, while 29 substances such as 2-propenyl benzoate were downregulated; compared with the blank control group, the (Z)-ethyl pentadec-9-enoate content increased by 43 fold and the diisobutyl phthalate content increased by 69.6 fold. In the FUBL10 group, 6 substances including 1,7-diazabicyclo[2.2.1]heptane were upregulated, and 13 substances such as picolinamide were downregulated; compared with the blank control group, the Diisobutyl phthalate content increased by 11.7 fold and the ylangene content increased by 11 fold. In summary, compared with the blank control group, the flavor metabolites in the FUBL1 and FUBL3 groups exhibited more pronounced differential changes. We speculate that when only the fortified fermentation strain was altered while all other conditions remained identical, FUBL1 and FUBL3 exerted a greater influence on the flavor compounds of rice-flavor Baijiu, whereas FUBL2 had the smallest impact.

The effects of the four *Bacillus velezensis* strains on the flavor metabolites of rice-flavor Baijiu exhibited obvious strain specificity. FUBL1 and FUBL3 induced 46 and 65 significantly differential metabolites, respectively, whereas FUBL2 and FUBL10 affected only 15 and 19 significantly differential flavor metabolites, respectively, and none of these metabolites were individually annotated as flavor compounds. Among the significantly upregulated flavor compounds, those unique to FUBL1 were 5-methyl-2-thiophenecarboxaldehyde (Feng et al., 2023) and 1-ethylpropyl acetate; the former exhibits a woody, sweet aroma reminiscent of almond and cherry, while the latter provides a fresh, sweet fruity note of apple and banana, both being desirable flavor contributors in Baijiu. The compounds unique to FUBL3 were Ethyl 2-methyl-2-butenoate, (−)-(E)-*β*-santalol, Benzyl benzoate, trans-Carveol and 1-Chloro-octadecane. (−)-(E)-β-Santalol possesses a natural sandalwood aroma (Delasalle et al., 2014), and ethyl 2-methyl-2-butenoate offers fresh fruity and floral notes (Jia et al., 2021); these substances play an important role in shaping the flavor of rice-flavor Baijiu. Unique to FUBL10 were two isomers of butanediol. Notably, the study revealed that several significantly down-regulated differential metabolites serve as precursors of the up-regulated metabolites, which explains the interdependence between the increase and decrease of flavor compounds. For instance, 1-butanol-2-methyl acetate can undergo transesterification with alcohols to form ethyl 2-methyl-2-butenoate, which was significantly upregulated among the flavor compounds; 2-propenyl benzoate undergoes transesterification to afford benzyl benzoate (Johnson et al., 2017); and the formation of N-(3,4-dimethoxybenzyl)-2-thiazolamine was also associated with 2-acetylthiazole (Wang et al., 2021). Collectively, these results further indicate that *Bacillus velezensis* may influence the synthesis and transformation of relevant flavor metabolites either through direct metabolism or through indirect modulation of the microbial community structure within the fermentation environment (Yang et al., 2022).

### 3.3 Analysis of the flavor profiles of differential metabolites

**Figure 3:**
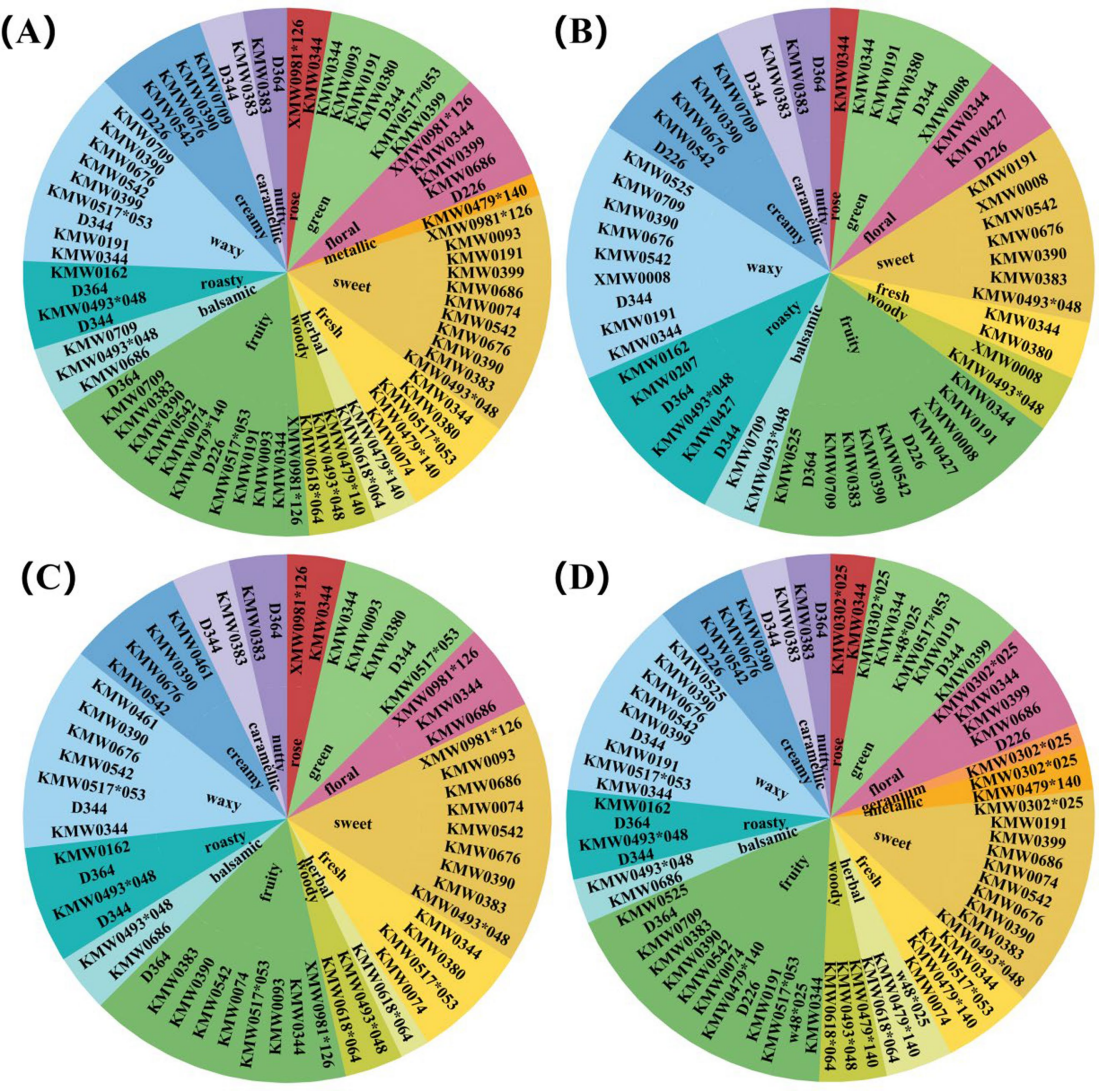
Flavor wheel of differential metabolites. **(A)** FUBL1; **(B)** FUBL2; **(C)** FUBL3; **(D)** FUBL10. The innermost ring shows the sensory flavor characteristics annotated to the differential metabolites of each group. The outermost ring represents the metabolite codes defined by mass-to-charge ratio (m/z) and retention time (RT) as determined by HS-SPME-GC–MS. See Research data for detailed compound information.

Based on the semi-quantitative results obtained by HS-SPME-GC–MS, we annotated the odor characteristics of the flavor compounds by consulting databases such as FooDB,^4^ TGSC,^5^ The Odour Database,^6^ Food Flavor Lab,^7^ and the relevant literature (Zhu et al., 2015; Wang et al., 2022; Fan and Xu, 2011; Gong et al., 2026). The relative odor activity value (rOAV) was calculated according to the established method. Compounds with rOAV ≥ 1 were selected to construct a flavor wheel (Figure 3); these compounds are considered to contribute to the flavor composition of Baijiu.

The results showed that the substances contributing most to the flavor were mainly terpenoids, esters, and heterocyclic compounds, along with a small number of alcohols, ketones. Terpenoids typically exhibit herbal, vanilla, and woody aroma characteristics (Wang et al., 2016). They serve as important aroma-active constituents in alcoholic beverages and also act as significant bioactive factors in Baijiu, possessing physiological effects such as analgesic, antibacterial, and antitumor activities (Fan and Xu, 2013). Esters mainly provide Baijiu with fruity, floral, sweet, and creamy flavor notes. Differences in their types and concentrations are critical factors that shape the distinct characteristics of different Baijiu (Wei et al., 2020). For example, ethyl hexanoate exhibits a pineapple-like aroma and contributes sweetness as well as a refreshing sensation (Xu et al., 2022), while ethyl butanoate has an odor reminiscent of pineapple and banana (Xu et al., 2020). Overall, the aroma profile and style of Baijiu are formed through the synergistic interactions of multiple complex sensory flavor components.

Compared with the blank group, the number of flavor compounds with rOAV ≥ 1 in the fermented grains increased in all enhanced fermentation groups (see Supplementary material: Key Flavor Compounds Shaping the Flavor Expression of Body). Among them, the FUBL1 group exhibited the highest number (21), followed by the FUBL10 group (19), the FUBL3 group (18), and the FUBL2 group (16). Notably, regarding key characteristic flavor compounds, the FUBL3 group significantly upregulated the contents of farnesylacetaldehyde and 4-vinylguaiacol, whereas the FUBL1 group specifically accumulated 4-vinylguaiacol. No such significantly upregulated flavor compounds were detected in the FUBL2 or FUBL10 groups. 4-Vinylguaiacol imparts woody, roasty, sweet, and balsamic-like notes to the liquor body. Based on the analysis of group-specific flavor components, among the flavor compounds with rOAV ≥ 1 and higher than those in the blank group, the FUBL2 group was characterized by 3-mercaptohexyl acetate, methyl furfuryl thiol, and isophorone; the FUBL3 group by farnesylacetaldehyde and nonanoic acid; and the unique compound in the FUBL10 group was rose oxide. No specific unique component was identified in the FUBL1 group, yet its overall flavor skeleton was more complete. Notably, ethyl decanoate, ethyl myristate, ethyl octanoate, 2-methyl-3-ethoxypyrazine, 1-nonanol, (E, E)-2,4-undecadienal, maple furanone, and dimethyl trisulfide were common to all four enhanced fermentation groups, suggesting that the introduction of *Bacillus velezensis* may universally elevate the levels of these flavor compounds. Further clustering analysis based on the flavor profiles (Figure 3) revealed significant differences in the aroma profiles among the groups. In the rose, geranium, and floral notes, the FUBL1 and FUBL10 groups exhibited the richest composition of volatile compounds, whereas the FUBL2 group displayed the most meager profile.

Within the sweet and fruity notes, besides the esters and pyrazines common to all four groups—such as ethyl decanoate, ethyl octanoate, ethyl myristate, and 2-methyl-3-ethoxypyrazine—ethyl 2-methylbutyrate and phenethyl isovalerate were shared by FUBL1 and FUBL3, while decanoic acid was shared by FUBL2 and FUBL10. Among the roasty character-impact flavor compounds, FUBL2 exhibited the richest profile; its unique constituents, 3-mercaptohexyl acetate and methyl furfuryl thiol, together with compounds common to all groups such as 2-methyl-3-ethoxypyrazine, jointly shaped the roasty aroma profile. Furthermore, in the caramellic and nutty flavor characteristics, maple furanone, (E, E)-2,4-undecadienal, and 2-methyl-3-ethoxypyrazine were common to all four fortified fermentation groups.

Integrating PCA, OPLS-DA modeling, and rOAV-based flavor activity evaluation, we demonstrated that the four *Bacillus velezensis* strains exhibited pronounced strain specificity in modulating the flavor metabolites of rice-flavor Baijiu. Using the number of significantly differential metabolites, the extent of increase in key aroma-active compounds, and the complexity of the aroma profile as comparative criteria, strains FUBL1 and FUBL3 showed clear superiority in both flavor enhancement and compound diversity. The FUBL1 group possessed the highest count (21) of flavor compounds that simultaneously satisfied rOAV ≥ 1 and exceeded the levels in the CK group; in particular, key aroma compounds such as benzyl salicylate and 4-vinylguaiacol were markedly elevated, leading to a more comprehensive overall flavor backbone. The FUBL3 group influenced the formation of the largest number of significantly differential metabolites (65) and specifically accumulated characteristic aroma compounds, including benzyl benzoate, ethyl 2-methyl-2-butenoate, and (−)-(E)-*β*-santalol, thereby significantly enriching the aromatic layers of the Baijiu across woody, roasty, and fruity dimensions. In contrast, the FUBL2 and FUBL10 groups yielded relatively fewer differential metabolites and lower richness of flavor-active compounds, resulting in only limited improvement of the flavor system.

### 3.4 Whole-genome profiles of strains FUBL1 and FUBL3

Through investigation of the effects of enhanced fermentation with *Bacillus velezensis* on flavor compounds in rice-flavor Baijiu, it was found that the FUBL1 and FUBL3 groups exhibited a larger number of significantly differential flavor compounds and superior enhancement effects, particularly in promoting the biosynthesis of terpenoids and esters. Therefore, the reasons why FUBL1 and FUBL3 influence the formation of terpenoids, esters, and other flavor compounds were analyzed in detail. To further elucidate the genetic mechanisms underlying these phenotypic differences, whole-genome sequencing and comparative genomic analysis of *B. velezensis* FUBL1 and FUBL3 were performed. By constructing bacterial genome framework maps, we systematically investigated the similarities and differences between the two strains with respect to key functional genes involved in terpenoid precursor biosynthesis, esterification reactions, associated metabolic pathways. This work clarifies, at the molecular level, the genetic basis for the differential accumulation of flavor compounds in rice-flavor Baijiu and provides a theoretical foundation for subsequent targeted breeding and fermentation regulation.

**Figure 4:**
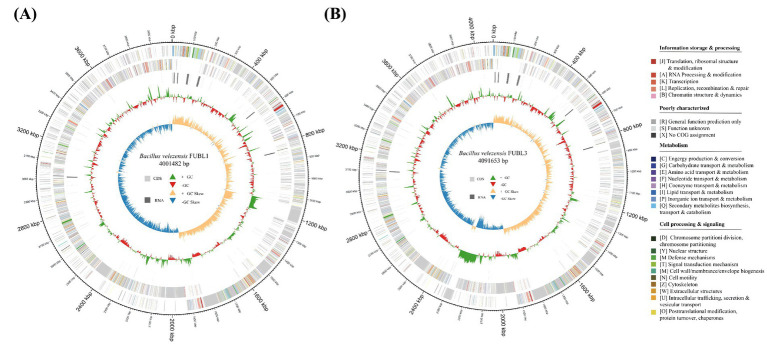
Whole-genome circular maps. **(A)** *B. velezensis* FUBL1. **(B)** *B. velezensis* FUBL3.

To elucidate the genetic basis underlying the differential efficacy of *B. velezensis* FUBL1 and FUBL3 in enhancing the flavor of rice-flavor Baijiu, this study first performed whole-genome sequencing of the two strains. The official accession number for FUBL1 is NMDC60316432.^8^ The official accession number for FUBL3 is NMDC60316433.^9^ As shown in Figure 4 (see Supplementary Figure 1), the genome of FUBL1 is 4,001,482 bp in length with a GC content of 46.49%, and harbors 3,887 predicted protein-coding sequences (CDSs) that account for 89.79% of the total genome length. The genome of FUBL3 is slightly larger, at 4,091,653 bp with a GC content of 46.34%, and harbors 4,136 predicted CDSs, representing 88.51% of the genome length. Both strains contain 86–87 tRNA genes and 9 complete copies of the rRNA gene cluster (each consisting of 5S, 16S, and 23S rRNA genes), suggesting similar basal capacities for protein synthesis. Nevertheless, the differences in genome size and total gene number preliminarily indicate structural divergence in the genetic content of the two strains. To further investigate whether this divergence is directly associated with their flavor metabolic phenotypes, we will subsequently conduct in-depth comparative genomics and functional annotation analyses based on the genomic framework established here.

### 3.5 Gene functional annotation of the strains using the KEGG database

To elucidate the genetic differences in flavor metabolic potential between strains FUBL1 and FUBL3, we performed annotation and comparative analysis of the metabolic pathway genes of the two strains based on alignment with the KEGG database. The results showed that, in flavor-associated pathways such as terpenoid precursor synthesis/terpenoid biosynthesis, esterification, and ester precursor synthesis, FUBL1 and FUBL3 shared 15 genes related to terpenoid synthesis, exemplified by 1-deoxy-D-xylulose-5-phosphate synthase (*dxs*, K01662), 1-deoxy-D-xylulose-5-phosphate reductoisomerase (*dxr*, K00099), and 2-C-methyl-D-erythritol 4-phosphate cytidylyltransferase (*ispD*, K00991); and 20 genes related to ester precursor synthesis, exemplified by acetyl-CoA acyltransferase (*fadA*, K00632), long-chain acyl-CoA synthetase (*fadD*, K01897), and fatty-acyl-CoA synthase (K00666). Furthermore, FUBL3 possessed 2 unique terpenoid synthesis-related genes: citronellol/citronellal dehydrogenase (*atuB*, K13774) and flavin prenyltransferase (*ubiX* / *bsdB* / *PAD1*, K03186).

**Figure 5:**
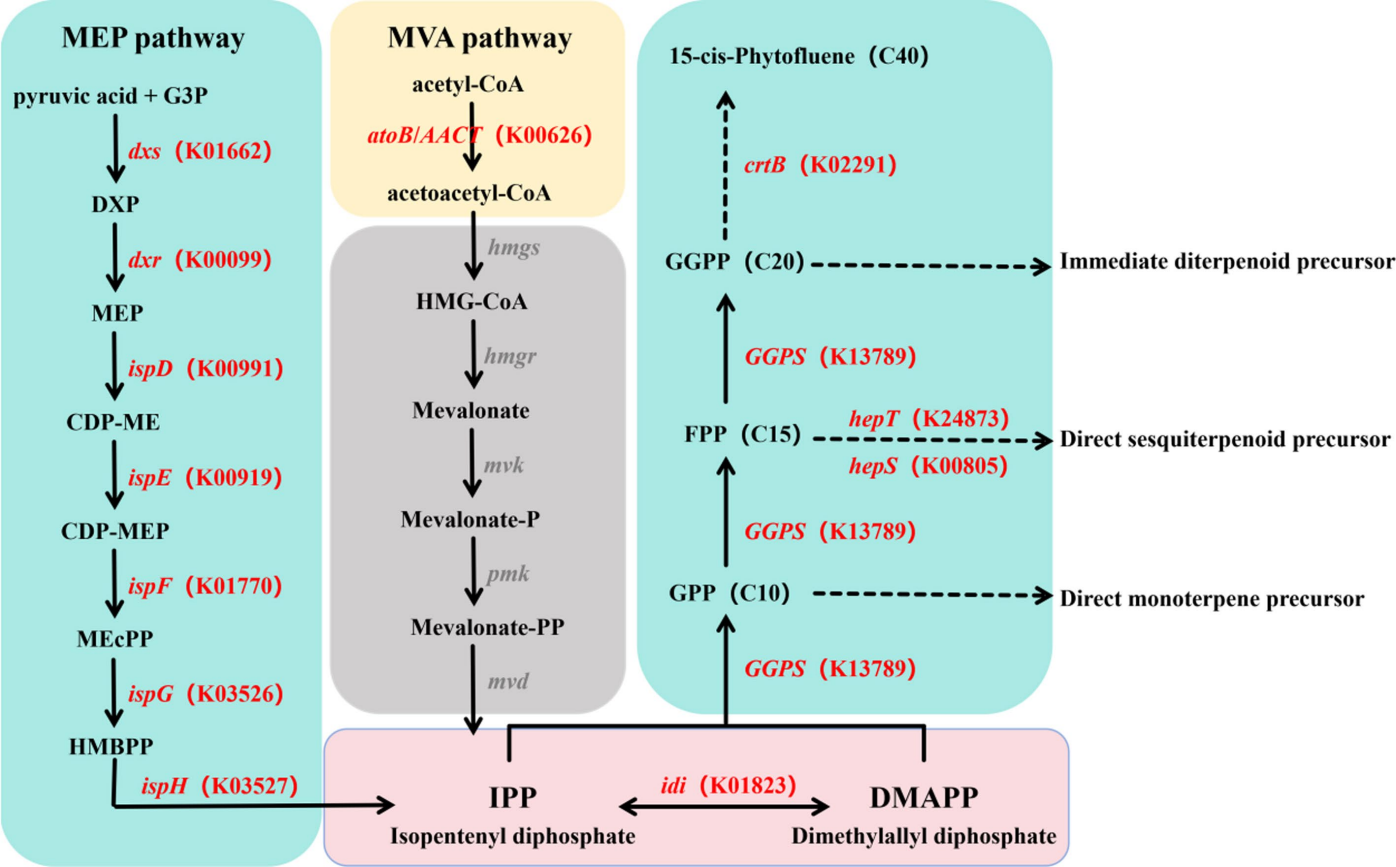
Pathways for microbial synthesis of terpenoids. Cyan and red squares indicate terpenoid biosynthetic pathways annotated in both FUBL1 and FUBL3. Yellow squares indicate the first key enzyme of the pathway annotated in FUBL1 and FUBL3. Gray squares indicate that these enzymes and genes are not annotated in the genomes of FUBL1 and FUBL3. Black text denotes relevant metabolites: G3P: glyceraldehyde-3-phosphate; DXP: 1-deoxy-D-xylulose 5-phosphate; MEP: 2-C-methyl-D-erythritol 4-phosphate; CDP-ME: 4-(cytidine 5′-diphospho)-2-C-methyl-D-erythritol; CDP-MEP: 2-phospho-4-(cytidine 5′-diphospho)-2-C-methyl-D-erythritol; MEcPP: 2-C-methyl-D-erythritol 2,4-cyclodiphosphate; HMBPP: 1-hydroxy-2-methyl-2-butene-4-diphosphonic acid; IPP: isopentenyl diphosphate; DMAPP: dimethylallyl diphosphate. Red text denotes relevant enzymes or genes: (*dxs*, K01662): 1-deoxy-D-xylulose-5-phosphate synthase; (*dxr*, K00099): 1-deoxy-D-xylulose-5-phosphate reductoisomerase; (*ispD*, K00991): 2-C-methyl-D-erythritol 4-phosphate cytidylyltransferase; (*ispE*, K00919): 4-diphosphocytidyl-2-C-methyl-D-erythritol kinase; (*ispF*, K01770): 2-C-methyl-D-erythritol 2,4-cyclodiphosphate synthase; (*ispG*, K03526): (E)-4-hydroxy-3-methylbut-2-enyl-diphosphate synthase; (*ispH*, K03527): 4-hydroxy-3-methylbut-2-en-1-yl diphosphate reductase; (*idi*, K01823): isopentenyl-diphosphate Delta-isomerase; (*atoB*/*AACT*, K00626): acetyl-CoA C-acetyltransferase; (*GGPS*, K13789): geranylgeranyl diphosphate synthase; (*hepT*/*hepS*, K24873/K00805): heptaprenyl diphosphate synthase; (*crtB*, K02291): 15-cis-phytoene synthase.

As shown in Table 1 and Figure 5 (see Supplementary Figure 1; Supplementary Table 1), in terms of terpenoid biosynthesis, the genes annotated in FUBL1 and FUBL3 encode nearly all the core enzymes of the methylerythritol phosphate (MEP) pathway, indicating that both strains possess a nearly complete suite of core MEP pathway enzymes and are capable of synthesizing the terpenoid precursors isopentenyl diphosphate (IPP) and dimethylallyl diphosphate (DMAPP). This explains why enhanced fermentation with *Bacillus velezensis* significantly increases terpenoid flavor compounds in rice-flavor Baijiu. Furthermore, a series of genes encoding enzymes responsible for the synthesis of complex terpenoids were also identified, such as heptaprenyl diphosphate synthase components (K24873 / K00805), which catalyze the conversion of farnesyl diphosphate (FPP) and four molecules of IPP into heptaprenyl diphosphate (Burtchett et al., 2025); geranylgeranyl diphosphate synthase (*GGPS*, K13789), a key enzyme for the synthesis of geranyl diphosphate (GPP) and geranylgeranyl diphosphate (GGPP)—GPP serving as the precursor for monoterpenoids and GGPP as the direct precursor for diterpenoids and tetraterpenoids; and 15-cis-phytoene synthase (*crtB*, K02291), which converts GGPP to phytoene (Gou et al., 2024). We found that, compared with FUBL1, FUBL3 harbors more genes associated with terpene biosynthesis. Specifically, FUBL3 was uniquely annotated with genes directly involved in terpene biosynthesis, including citronellol/citronellal dehydrogenase (*atuB*, K13774) and flavin prenyltransferase (*ubiX* / *bsdB* / *PAD1*, K03186). Citronellol/citronellal dehydrogenase directly participates in the microbial production of citronellol and citronellal—oxygenated monoterpene derivatives that serve as important constituents shaping the layered flavor profile of Baijiu (Lee et al., 2025). This is one reason why FUBL3 can produce a more diverse array of terpenoids than FUBL1, which aligns with the earlier finding that FUBL3 exhibited the greatest diversity of annotated terpenoid metabolites. Notably, although an acetyl-CoA C-acetyltransferase gene (*atoB* / *AACT*, K00626)—encoding the enzyme that catalyzes the first step of the mevalonate (MVA) pathway for terpenoid backbone biosynthesis—was annotated in the genomes of FUBL1 and FUBL3, no downstream genes of the complete MVA pathway (e.g., *hmgs*, *hmgr*, *mvk*, *pmk*, *mvd*) were identified. This indicates that the primary function of this gene is not terpenoid backbone biosynthesis. Taken together, these findings suggest that FUBL1 and FUBL3 possess the potential to synthesize complex terpenoids, thereby providing rice-flavor Baijiu with a rich array of terpenoid flavor metabolites.

**Table 1:** Genes involved in terpenoids biosynthesis were annotated using the KEGG database.

Classification	KEGG number	Description	Genes
Shared genes	K01662	1-deoxy-D-xylulose-5-phosphate synthase	*dxs*
	K00099	1-deoxy-D-xylulose-5-phosphate reductoisomerase	*dxr*
	K00991	2-C-methyl-D-erythritol 4-phosphate cytidylyltransferase	*ispD*
	K00919	4-diphosphocytidyl-2-C-methyl-D-erythritol kinase	*ispE*
	K01770	2-C-methyl-D-erythritol 2,4-cyclodiphosphate synthase	*ispF*
	K03526	(E)-4-hydroxy-3-methylbut-2-enyl-diphosphate synthase	*ispG*
	K03527	4-hydroxy-3-methylbut-2-en-1-yl diphosphate reductase	*ispH*
	K01823	isopentenyl-diphosphate Delta-isomerase	*idi*
	K00626	acetyl-CoA C-acetyltransferase	*atoB / ACAT*
	K13789	geranylgeranyl diphosphate synthase	*GGPS*
	K02291	15-cis-phytoene synthase	*crtB*
	K00805	heptaprenyl diphosphate synthase component 1	*hepS*
	K24873	heptaprenyl diphosphate synthase component 2	*hepT*
	K23138	cytochrome P450 family 109	*CYP109*
	K14338	cytochrome P450 / NADPH-cytochrome P450 reductase	*cypD/E*
FUBL3	K13774	citronellol/citronellal dehydrogenase	*atuB*
	K03186	flavin prenyltransferase	*ubiX / bsdB / PAD1*

In the genomes of *Bacillus velezensis* FUBL1 and FUBL3, comparative analysis against existing databases (e.g., KEGG, UniProt) did not identify any classical ester synthases with high homology, such as alcohol acyltransferase (AAT) and wax ester synthase (WS / DGAT; Zhou et al., 2021; Kuribayashi et al., 2026). However, the biosynthesis of esters follows the biochemical principle of “precursor activation”: free fatty acids (FFAs) must first be converted into activated acyl-CoAs, catalyzed by acyl-CoA synthetases, to serve as acyl donors in esterification reactions (Saha et al., 2024; Xiao et al., 2023). The acetyl-CoA synthetase (*ACS*, K01895) annotated in this study is responsible for converting acetate to acetyl-CoA and is hypothesized to provide the direct substrate for a putative acetate ester biosynthesis module; the long-chain acyl-CoA synthetase (*fadD*, K01897) generates long-chain fatty acyl-CoAs, which may supply activated precursors for the synthesis of long-chain fatty acid ethyl esters or lipopeptide compounds. Therefore, although classical ester synthases were not directly identified, the presence of these acyl-CoA synthetases constitutes the upstream genetic basis for the potential flavor ester biosynthesis capability of these strains.

As shown in Table 2 (see Supplementary Table 2), genomic analysis revealed that FUBL1/FUBL3 harbor a complete set of genes encoding the branched-chain amino acid degradation enzyme system (K00166 / K00167 and K09699, annotated as 2-oxoisovalerate dehydrogenase complex components). This enzyme system catalyzes the conversion of branched-chain amino acids into branched-chain acyl-CoA, thereby supplying potential precursor substrates for the synthesis of branched-chain fatty acid esters (Krink-Koutsoubelis et al., 2018). Triacylglycerol lipase (K01046) hydrolyzes triacylglycerols to release free fatty acids, providing key precursors for the formation of flavor esters. Acetyl-CoA carboxylase (K01962 / K01963) catalyzes the production of malonyl-CoA and functions as the rate-limiting enzyme in fatty acid synthesis; its activity determines the preferred chain length of the fatty acids synthesized by the strain, thus providing the carbon skeleton for the biosynthesis of fatty acid precursors of flavor esters. Furthermore, the genes encoding acetate kinase (K00925) and phosphate acetyltransferase (K00625) constitute the pathway for converting free acetate to acetyl-CoA, while acyl-CoA thioester hydrolase (K07107) mediates the reverse hydrolysis. Together, they allow dynamic interconversion between acetate and acetyl-CoA (Swarbrick et al., 2020; Mio et al., 1999). This pathway dynamically modulates the acetyl-CoA flux and indirectly controls the synthesis of flavor esters such as ethyl acetate at the metabolic level, representing a core link in acetate utilization and flavor precursor synthesis during microbial fermentation.

**Table 2:** Genes involved in ester synthesis were annotated using the KEGG database.

Classification	KEGG number	Description	Genes
Shared genes	K00632	acetyl-CoA acyltransferase	*fadA*
	K01066	acetyl esterase	*aes*
	K07107	acyl-CoA thioester hydrolase	*ybgC*
	K23978	isoamyl acetate-hydrolyzing esterase	*IAH1*
	K00625	phosphate acetyltransferase	*pta*
	K01897	long-chain acyl-CoA synthetase	*fadD*
	K01895	acetyl-CoA synthetase	*acs*
	K09699	2-oxoisovalerate dehydrogenase E2 component (dihydrolipoyl transacylase)	*DBT, bkdB*
	K00166	2-oxoisovalerate dehydrogenase E1 component alpha subunit	*BCKDHA / bkdA1*
	K00167	2-oxoisovalerate dehydrogenase E1 component beta subunit	*BCKDHB / bkdA2*
	K00925	acetate kinase	*ackA*
	K01963	acetyl-CoA carboxylase subunit beta / acetyl-CoA carboxylase carboxyl transferase subunit beta	*accD*
	K01962	acetyl-CoA carboxylase subunit alpha / acetyl-CoA carboxylase carboxyl transferase subunit alpha	*accA*
	K00140	malonate-semialdehyde dehydrogenase(acylating)	*mmsA / iolA / ALDH6A1*
	K00666	fatty-acyl-CoA synthase	
	K09458	3-oxoacyl-[acyl-carrier-protein] synthase II	*fabF / OXSM / CEM1*
	K01046	triacylglycerol lipase	*Lip / TGL2*
	K03928	carboxylesterase	*yvaK*
	K00954	pantetheine-phosphate adenylyltransferase	*coaD / kdtB*
	K00004	(R, R)-butanediol dehydrogenase / meso-butanediol dehydrogenase / diacetyl reductase	*BDH / butB*
	K13979	alcohol dehydrogenase (NADP+)	*yahK*
	K13953	alcohol dehydrogenase, propanol-preferring	*adhP*
	K07250	4-aminobutyrate aminotransferase / (S)-3-amino-2-methylpropionate transaminase / 5-aminovalerate transaminase	*gabT*
	K11358	aspartate aminotransferase	*yhdR*
	K10907	aminotransferase	
	K00128	aldehyde dehydrogenase (NAD+)	*ALDH*
	K19955	alcohol dehydrogenase	*adh2*

Acetyl esterase (K01066) belongs to the *α*/*β*-hydrolase superfamily and possesses reversible catalytic properties. Previous studies have demonstrated that acetyl esterase can catalyze transesterification reactions in aqueous systems, suggesting that reversible catalytic capability may be a more general feature of the α/β-hydrolase superfamily. Theoretically, during the early stage of fermentation when the water activity is high, this enzyme primarily functions as a hydrolase, degrading esters; in the late fermentation stage, as the ethanol concentration increases and the water activity decreases, the reaction equilibrium may shift toward esterification or transesterification, thereby promoting the synthesis of short-chain fruity esters such as ethyl acetate. However, this function has not been directly verified experimentally in *Bacillus velezensis*. Based on the above theory and catalytic characteristics, we hypothesize that acetyl esterase (K01066) plays a role in dynamically regulating the balance of esters within the metabolic network of *Bacillus velezensis*. Isoamyl acetate-hydrolyzing esterase (*IAH1*, K23978) exhibits typical reversible catalytic properties. It has been reported to catalyze the synthesis of isoamyl acetate under low water activity, non-aqueous conditions (Torres et al., 2009). During winemaking, *IAH1* has been demonstrated to possess hydrolytic activity and to serves as a key regulator of acetate ester balance (Wang Z. et al., 2024).

## 4 Discussion

*Bacillus* is an important genus in Baijiu Jiuqu (fermentation starter) and plays roles in substrate degradation, flavor synthesis, and microecological regulation during the brewing process (Zhu et al., 2025). In this study, we employed an integrated strategy combining flavor metabolomics and genomics to systematically evaluate the differential impacts of four *Bacillus velezensis* strains on the flavor metabolites of rice-flavor Baijiu. Although all strains belong to *B. velezensis*, their regulatory effects on the volatile metabolic profile of fermented grains exhibited pronounced strain specificity. Among them, strains FUBL1 and FUBL3 showed the most prominent enhancement effects, significantly upregulating a wider variety of terpenoids and esters and creating a more complex and multi-layered flavor profile (e.g., fruity, floral, sweet, and woody notes), whereas the effects of FUBL2 and FUBL10 were relatively limited. Therefore, when utilizing functional microorganisms for flavor modulation, precise screening at the strain level is crucial, as generalized functional inferences at the genus or species level may introduce biases.

The shared genomic features of FUBL1 and FUBL3 provide key insights into why they are highly efficient flavor-synthesizing strains. Comparative genomic analysis revealed that both strains possess a complete core metabolic network associated with the synthesis of flavor precursors. They harbor a nearly complete gene cluster of the methylerythritol phosphate (MEP) pathway (Vranová et al., 2013), which provides the genetic basis for efficient synthesis of the terpenoid backbones, isopentenyl diphosphate (IPP) and dimethylallyl diphosphate (DMAPP), thereby markedly increasing the diversity of terpenoids in fermented grains. Furthermore, both strains encode a full suite of enzymes responsible for acetyl-CoA generation through to medium- and long-chain fatty acyl-CoA elongation, ensuring a sustained supply of activated acyl donors of various chain lengths for esterification (Chen et al., 2025). This likely represents the primary cause of the widespread increase in total ester content (particularly long-chain fatty acid ethyl esters) observed in the bioaugmented groups. FUBL1 and FUBL3 also exhibit differences in shaping specific flavor profiles, which arise from subtle variations in their genomes. FUBL3 is more proficient in terpenoid derivatization. Its unique citronellol/citronellal dehydrogenase gene (K13774) may directly drive the synthesis of oxygenated monoterpenes (e.g., citronellol), thus not only enriching terpenoid diversity but also contributing a more elegant floral and fruity aroma. This is consistent with the more pronounced floral note in the flavor wheel of the FUBL3 group. The genomic composition of genes related to ester synthesis is largely similar between the two strains. It is hypothesized that differences in the copy numbers of key ester synthase genes lead to FUBL1 specifically accumulating characteristic metabolites such as 5-methyl-2-thiophenecarboxaldehyde (sweet almond aroma) and 1-ethylpropyl acetate (apple aroma), ultimately resulting in a differentiated flavor metabolite profile.

The study also found that, in fortified fermentation, strains FUBL1, FUBL2, FUBL3, and FUBL10 all significantly increased the contents of terpenoids such as *β*-cubebene and *α*-pinene, with β-cubebene increasing by 63-fold, 33.5-fold, 81-fold, and 34-fold and α-pinene by 2.3-fold, 1.2-fold, 2.2-fold, and 1.2-fold, respectively. β-Cubebene and α-pinene are generally recognized as typical plant-derived terpenes, and classical terpene synthases responsible for their direct biosynthesis have not been reported in bacteria such as *Bacillus* species (Sales et al., 2020). We speculate that this phenomenon is closely associated with the saccharification-promoting properties of these four strains and the resulting microecological alterations in the fermentation system. Genomic analysis revealed that FUBL1 and FUBL3 were annotated to contain a large number of glycoside hydrolases (e.g., families GH1, GH11, and GH13; see Supplementary material 3). The extracellular enzymes secreted by FUBL1 and FUBL3 can act synergistically with molds in the Jiuqu to efficiently hydrolyze starch and structural polysaccharides in the rice substrate, thereby markedly accelerating the saccharification process (Cairns and Esen, 2010). Meanwhile, the intensified fermentation altered the original microbial community structure, promoting the growth of microorganisms with glycoside hydrolase capabilities or the secretion of their related enzyme systems. These enzymatic and microbial activities, in turn, hydrolyzed the bound plant-derived terpene precursors present in the raw material or Jiuqu, thereby releasing free volatile compounds such as α-pinene (Kfoury et al., 2024; De Beul et al., 2024). Therefore, the pronounced increase in the ultimately detected levels of β-cubebene, α-pinene, and related compounds most likely reflects the efficient liberation of bound aroma precursors rather than substantial *de novo* biosynthesis by the microorganisms themselves.

Notably, although FUBL1 and FUBL3 lack a complete mevalonate (MVA) pathway, the gene encoding acetyl-CoA C-acetyltransferase (*atoB* / *AACT,* K00626) is still annotated in their genomes. This indicates that the primary role of this gene in these strains is no longer terpenoid biosynthesis but rather the regulation of carbon metabolic flux in central metabolism. *AACT* catalyzes the formation of acetoacetyl-CoA, which serves as a precursor for the fatty acid elongation cycle, thereby providing the critical carbon skeleton for the abundant short-chain fatty acid esters synthesized by these strains. Moreover, this enzyme catalyzes a reversible reaction that acts as a key bidirectional switch for acetyl-CoA metabolism: when carbon sources are plentiful, it condenses acetyl-CoA to generate acetoacetyl-CoA, supplying the core backbone for the synthesis of fatty acids, esters, and polyketides; under carbon limitation, it cleaves acetoacetyl-CoA back to acetyl-CoA to replenish the acetyl-CoA pool, thereby maintaining tricarboxylic acid cycle flux and securing cellular energy supply (Bennett and Rudolph, 1995; Kayani et al., 2023).

In summary, through integrated metabolomic and genomic analyses, this study systematically elucidated the differential regulatory mechanisms by which different *Bacillus velezensis* strains modulate the flavor of rice-flavor Baijiu at both the metabolic phenotype level and genetic levels. Our findings confirm that genotypic variation among strains is the core determinant of their flavor-modulating efficacy and specificity, underscoring the necessity of precise strain screening and functional validation in the development and application of functional microorganisms. The results of this study provide crucial microbial resources and a theoretical foundation for constructing a stable, efficient, next-generation rice-flavor Baijiu brewing system capable of targeted flavor design.

## 5 Conclusion

In this study, an integrated multi-omics approach was employed to systematically elucidate the differential impacts and underlying molecular mechanisms of four *Bacillus velezensis* strains on flavor-associated metabolites in rice-flavor Baijiu. All four *Bacillus velezensis* strains significantly altered the volatile metabolic profile of the simulated fermentation system for rice-flavor Baijiu, but the effects on the types, quantities, and sensory characteristics of flavor compounds differed markedly among strains.

Among them, the enhancement achieved by FUBL1 and FUBL3 was the most pronounced. The FUBL1 group contained the highest number of flavor compounds (21) with rOAV ≥ 1 that also exceeded the levels in the CK group; key aroma compounds such as benzyl salicylate and 4-vinylguaiacol were significantly increased, resulting in the most complete overall flavor skeleton. The FUBL3 group influenced the formation of the largest number of significantly differential metabolites (65) and specifically accumulated flavor compounds including benzyl benzoate, ethyl 2-methyl-2-butenoate, and (−)-(E)-*β*-santalol. FUBL2 and FUBL10 imparted fruity, sweet, and roasty profiles to the liquor, but their impacts were relatively limited. Comparative genomic analysis revealed that both FUBL1 and FUBL3 possess complete pathways for the biosynthesis of terpenoid precursors (the MEP pathway) and ester precursors (acetyl-CoA metabolism, branched-chain amino acid degradation, and fatty acid synthesis and degradation). The citronellol/citronellal dehydrogenase uniquely identified in FUBL3 was directly associated with its capacity to produce a richer diversity of terpenoids. This study validates the effectiveness of *Bacillus velezensis* as a functional microorganism for enhancing the flavor of rice-flavor Baijiu and, more importantly, reveals that strain specificity is a decisive factor in determining the outcome of flavor modulation. These findings provide solid theoretical support and specific strain resources for the genomics-guided precision breeding of functional strains tailored for Baijiu fermentation and for the targeted application of these strains.

## Data Availability

The datasets presented in this study can be found in online repositories. The names of the repository/repositories and accession number(s) can be found in the article/Supplementary material.

## Footnotes

1
https://www.kegg.jp/

2
https://nmdc.cn

3
www.r-project.org

4
https://foodb.ca

5
http://www.thegoodscentscompany.com

6
http://www.odour.org.uk/odour/index.html

7
http://foodflavorlab.cn/#/home

8
https://nmdc.cn/resource/genomics/genome/detail/NMDC60316432

9
https://nmdc.cn/resource/genomics/genome/detail/NMDC60316433
