# Supplementary material for: Flavor compounds regulation in rice-flavor Baijiu by Bacillus velezensis: a genetic and functional analysis
Source: Front Microbiol. 2026 Jul 24;17:1891609. doi: 10.3389/fmicb.2026.1891609 (PMC13447298; doi:10.3389/fmicb.2026.1891609)
Supplement: Supplementary file 1 [file Data_Sheet_1.docx]

**Supplementary Material**

**Figure 1.** Results of PCA and OPLS-DA models. (A) Principal component analysis (PCA). (B) OPLS-DA model analysis.

**Table 1.** Glycoside hydrolases possessed by the strain.

| Shared genes | K01220 | 6-phospho-beta-galactosidase | GH1 |
| --- | --- | --- | --- |
|  | K01222 | 6-phospho-beta-glucosidase | GH4 |
|  | K07406 | alpha-galactosidase |  |
|  | K01232 | maltose-6-phosphate glucosidase |  |
|  | K01179 | endoglucanase | GH5 |
|  | K01176 | alpha-amylase | GH13_28 |
|  | K01226 | trehalose-6-phosphate hydrolase | GH13_29 |
|  | K01187 | alpha-glucosidase | GH13_31 |
|  | K01182 | oligo-1,6-glucosidase | GH13_31 |
|  | K01218 | mannan endo-1,4-beta-mannosidase | GH26 |
|  | K15924 | glucuronoarabinoxylan endo-1,4-beta-xylanase | GH30_8 |
|  | K01193 | beta-fructofuranosidase | GH32 |
|  | K01212 | levanase |  |
|  | K06113 | arabinan endo-1,5-alpha-L-arabinosidase | GH43_4 |
|  | K01198 | xylan 1,4-beta-xylosidase | GH43_11 |
|  | K01209 | alpha-L-arabinofuranosidase | GH51 |
|  | K01224 | arabinogalactan endo-1,4-beta-galactosidase | GH53 |
|  | K00692 | levansucrase | GH68 |
|  | / | amylase | GH126 |
| FUBL1 | K01181 | endo-1,4-beta-xylanase | GH11 |
|  | / | endo-α-1,6-mannanase | GH76 |
| FUBL3 | K01809 | mannose-6-phosphate isomerase | GH1 |
|  | K01223 | 6-phospho-beta-glucosidase |  |
|  | / | endo-β-1,4-glucanase | GH6 |
|  | K15921 | arabinoxylan arabinofuranohydrolase | GH43 |
|  | / | sucrose b-2,1-fructosidase | GH100 |
|  | / | endo-β-1,4-mannanase | GH113 |

3. **An Excel file: Significantly differential flavor compounds screened using the criteria of |log₁₀(fold change)| ≥ 1 and p < 0.05.**
4. **An Excel file: Key Flavor Compounds Shaping the Flavor Expression of Body**
5. **An Excel file: Raw data obtained by HS-SPME-GC-MS**
